# Mucinous adenocarcinoma caused by cancerization from a ciliated multinodular papilloma tumor: A case report

**DOI:** 10.1111/1759-7714.13956

**Published:** 2021-04-03

**Authors:** Feng Chen, Fan Ren, Honglin Zhao, Xiaoqian Xu, Jun Chen

**Affiliations:** ^1^ Department of Lung Cancer Surgery Tianjin Medical University General Hospital Tianjin China; ^2^ Tianjin Key Laboratory of Lung Cancer Metastasis and Tumor Microenvironment Lung Cancer Institute, Tianjin Medical University General Hospital Tianjin China; ^3^ Health management center Tianjin Medical University General Hospital Tianjin China

**Keywords:** ciliated multinodular papilloma tumor, gene mutation, mucinous adenocarcinoma

## Abstract

Ciliated multinodular papilloma tumor (CMPT), a subtype of proximal bronchiolar adenoma (BA), is a rare mucin‐producing papillary tumor arising in the peripheral lung. The nature of CMPT is so far controversial. The hypothesis that CMPT is a precancerous lesion that can lead to mucinous adenocarcinoma requires further research. A 61‐year‐old man with a ground‐glass opacity (GGO) suspected to be lung adenocarcinoma in the right lower lobe of his lung underwent surgical treatment. Postoperative pathology suggested that the patient had mucinous adenocarcinoma caused by cancerization from CMPT. Targeted next‐generation sequencing (NGS) was utilized to detect driver mutations in tumor DNA. Among the identified mutated genes, there were regrettably no high frequency mutations. This report describes a case of mucinous adenocarcinoma caused by cancerization from CMPT, indicating that CMPT may be a neoplasm rather than a metaplastic process and provides histological evidence for the hypothesis that CMPT is a precancerous lesion of mucinous adenocarcinoma.

## Graphical abstract

This report describes a case of mucinous adenocarcinoma caused by cancerization from CMPT. This indicates CMPT may be a neoplasm rather than a metaplastic process and provides histological evidence for the hypothesis that CMPT is a precancerous lesion of mucinous adenocarcinoma

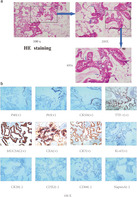

## INTRODUCTION

Ciliated multinodular papilloma tumor (CMPT) arises in the peripheral lung fields and is associated with the proliferation of ciliated and goblet cells and increased mucin production. CMPT was first reported by Ishikawa and Tsuchiya in 2002^1^ and has not yet been classified according to the 2015 World Health Organization (WHO) classification system.^2^ Given the partially overlapping morphology and similar genomic profiles, some researchers believe that the classic ciliated multinodular papilloma tumor (CMPT) is a subgroup of proximal bronchiolar adenoma (BA), which has a predominantly papillary architecture.^3^ Although no recurrent lesions arose in previously reported cases of CMPT, the exact nature of this tumor, whether it is benign or malignant, has so far not been established. Rare cases of CMPT harboring a component of well differentiated adenocarcinoma have been previously reported.^4^ However, the cancerization of CMPT is rare. Here, for the first time, we report a case diagnosed with mucinous adenocarcinoma caused by the cancerization of CMPT.

## CASE REPORT

**FIGURE 1:**
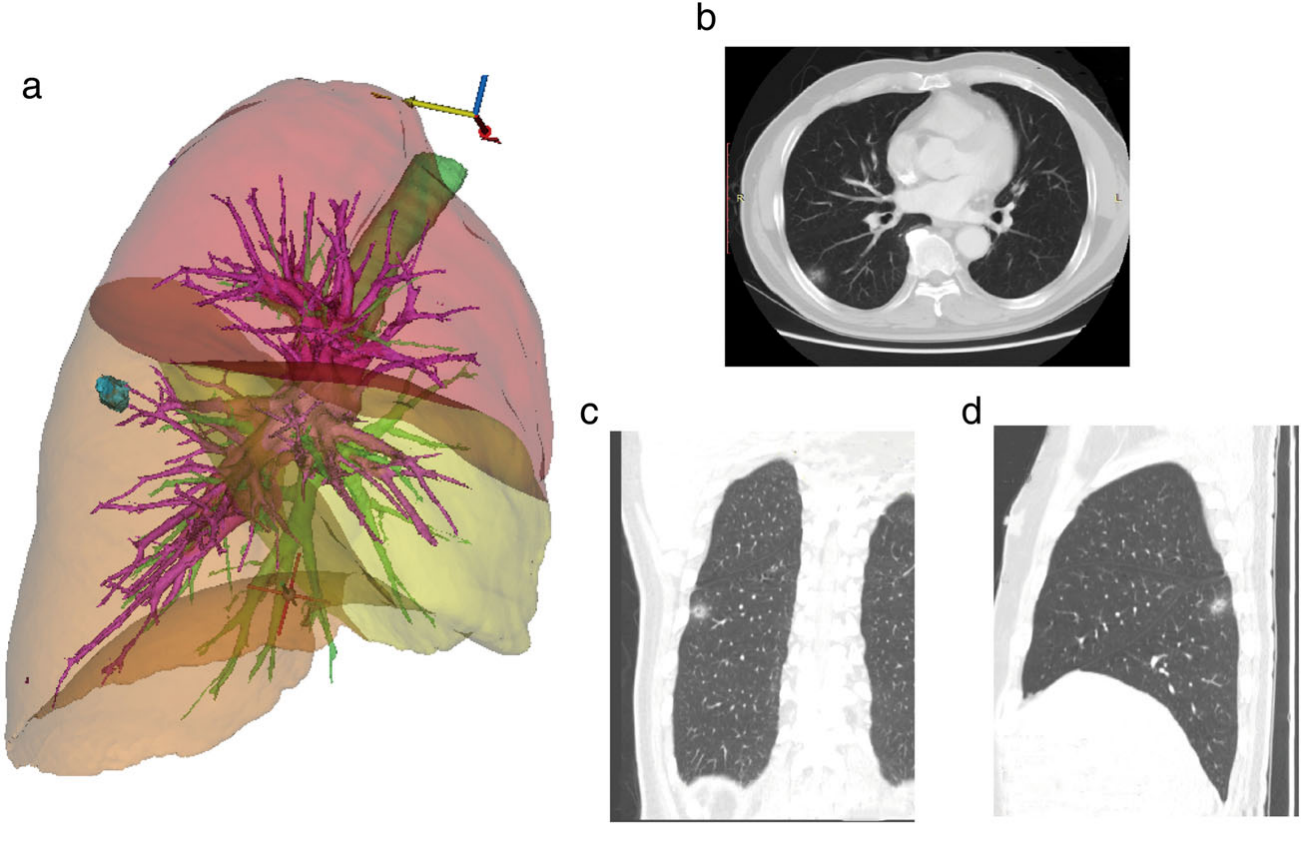
The CT scans of a ciliated multinodular papilloma tumor (CMPT). A 14 mm ground‐glass opacity (GGO) in the right lower lobe of the lung on computed tomography (CT) scans, adjoined to pleura, was resected via video‐assisted thoracoscopic surgery (VATS) and was eventually confirmed as mucinous adenocarcinoma caused by the cancerization of CPMT (proximal BA)

A 61‐year‐old man with a smoking index of 100 was admitted to our department for evaluation of a 14 mm ground‐glass opacity (GGO) in the right lower lobe of his lung, which had been an incidental finding six months previously (Figure 1). Laboratory investigations showed a neuron enolase level of 22.73 μg/l, although other index levels were normal. The nodule had a standardized uptake value of 1.30 on 18F‐fluorodeoxyglucose positron emission tomography (FDG‐PET) imaging. Based on these findings, lung cancer was suspected although a definite diagnosis could not be established based on radiological or clinical evaluation.

**FIGURE 2:**
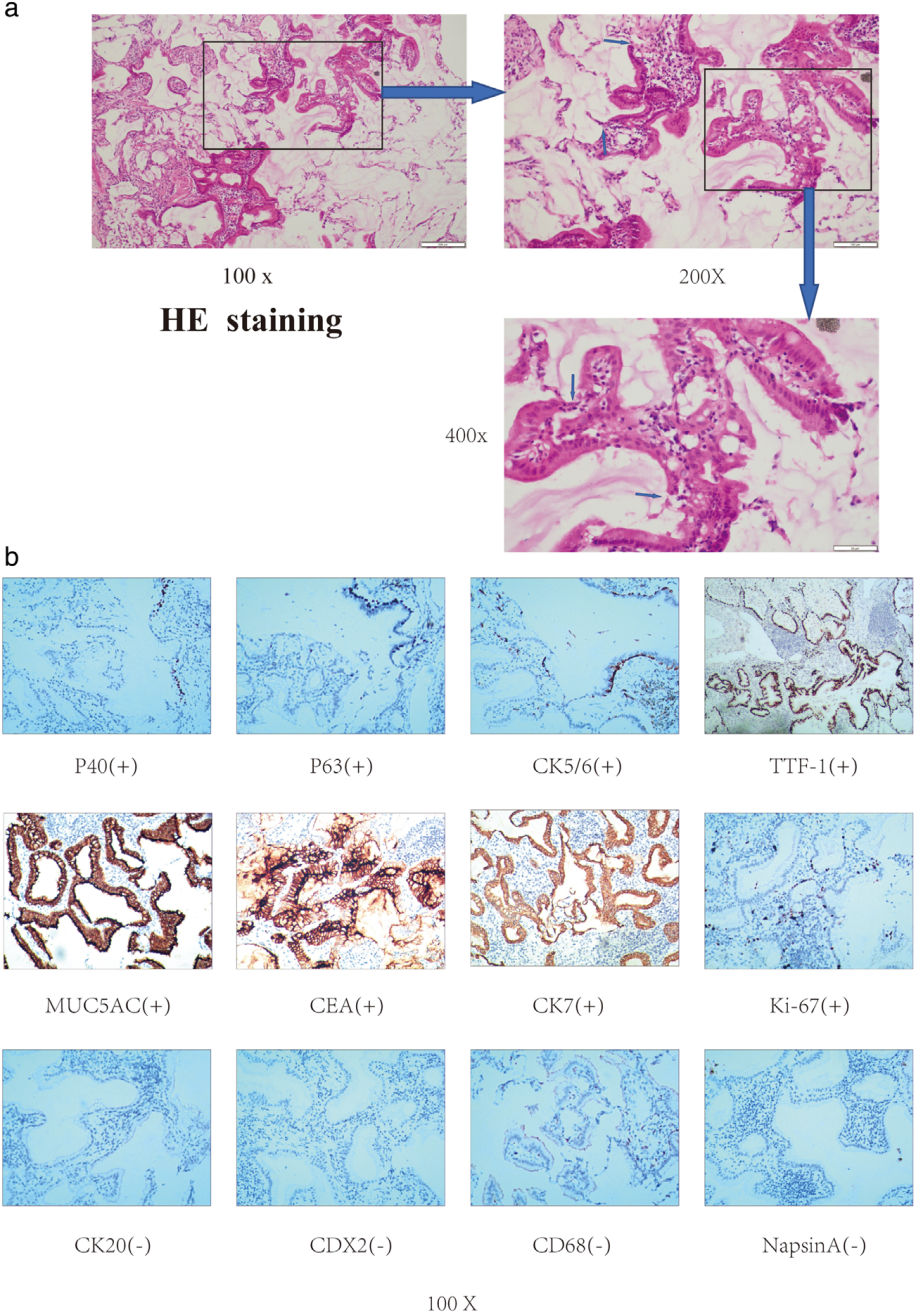
Hematoxylin & eosin (H&E) and immunohistochemical (IHC) staining results. (a) H&E staining. The arrow indicates a double‐layer cell structure composed of basal cells and ciliated cells in some areas, and the basal cells in some areas were discontinuous or even disappeared. (b) IHC staining shows diffuse positivity for thyriod transcription factor‐1 (TTF‐1) and partial positivity for mucin5AC (MUC5AC), carcinoembryonic antigen (CEA) and cytokeratin 7 (CK7) with a Ki‐67 index of approximately 6%–28% and negativity for cytokeratin 20 (CK20), caudal‐related homeobox transcription factor 2(CDX2), CD68 and aspartic proteinase napsin (Napsin A), P40, p63 and cytokeratin 5/6 (CK5/6) were expressed in the area where basal cells existed

Following the guidance of intraoperative frozen pathology, the patient underwent a right lower lobe resection via video‐assisted thoracoscopic surgery (VATS). Macroscopic examination of the resected lung parenchyma specimen showed a well circumscribed gray nodule measuring 10 × 18 × 15 mm. Pathology and hematoxylin & eosin (HE) staining showed that there was a double‐layer cell structure composed of basal and ciliated cells, which were typical of CMPT, but the basal cells in some areas were discontinuous or even disappeared, which was one of the reasons why we suspected cancerization (Figure 2). Confirmed by immunohistochemistry, the pathology of postoperative specimens suggested that the ground‐glass opacity was mucinous adenocarcinoma caused by the cancerization of CPMT (proximal BA).

To explore the underlying molecular mechanisms and their potential therapeutic relevance, we utilized targeted next‐generation sequencing (NGS) to detect driver mutations in tumor DNA (tDNA) (Genetron Health). A panel of 825 tumor‐related genes was subjected to NGS. The sequencing results identified 18 genes that each contained a single missense mutation (Table 1). No copy number variation or gene translocation events were detected. There was no high frequency mutation among the identified mutated genes.

**TABLE 1:** List of mutations

Gene name	Variant classification	cDNA change	Protein change	Exon num
EPHA2	Missense variant	c.2823C > A	p.Asn941Lys	16|17
MUTYH	Missense variant	c.673G > A	p.Val225Ile	9|16
XPC	Missense variant	c.1789C > T	p.Arg597Trp	9|16
TP63	Missense variant	c.2039A > G	p.Glu680Gly	14|14
EPHA5	Missense variant	c.1091C > T	p.Ala364Val	5|18
EPHA7	Missense variant	c.200C > T	p.Pro67Leu	3|17
EPHB4	Missense variant	c.2785G > A	p.Ala929Thr	16|17
SMARCA2	Missense variant	c.1330C > T	p.Arg444Cys	7|34
MAPK8IP1	Missense variant	c.1753G > A	p.Val585Ile	8|12
ERBB3	Missense variant	c.3119G > A	p.Arg1040Gln	25|28
HIF1A	Missense variant	c.542A > G	p.Lys181Arg	5|15
CHD2	Missense variant	c.3587C > A	p.Ala1196Asp	28|39
IGF1R	Missense variant	c.2827G > A	p.Ala943Thr	14|21
FZR1	Missense variant	c.133G > A	p.Gly45Arg	3|14
INSR	Missense variant	c.1874C > A	p.Pro625His	9|22
USHBP1	Missense variant	c.1603C > T	p.Arg535Trp	10|13
SMC1A	Missense variant	c.1864G > A	p.Val622Met	11|25
TAF1	Missense variant	c.3662G > A	p.Arg1221Gln	24|38

## DISCUSSION

Most cases of CMPT are peripheral solid, ground‐glass, or mixed solid nodules on radiological evaluation, and have previously been reported in patients from East Asian countries, particularly Japan.^5^, ^6^ However, the authors believe that this regional epidemiological difference is caused by the popularity of CT screening. The average size of most CMPT ranges from 9.1 to 11 mm and the growth speed is 0.49 mm/year which is similar to a benign lung tumor, and they appear as well circumscribed gray nodules with or without mucus on gross inspection.^7^ In terms of pathology, all tumors were composed of bilayered cellular proliferation with a continuous basal cell layer, highlighted by basal cell markers p40 and/or CK5/6.^3^ It could have been misdiagnosed as preinvasive lesions or invasive carcinoma, such as mucinous AIS and colloid adenocarcinoma at a low‐power view, particularly when referring to frozen section slides. However, on examination of the slides at a high‐power view, the ciliated and basaloid cells could be identified, thereby avoiding any misinterpretation.^8^ Therefore, CMPT is often a trap for a differential diagnosis of adenocarcinoma on frozen section slides. In this case, the frozen section revealed an epithelial tumor, which is a papillary growth with infiltration, and could not be distinguished from a malignant tumor.

The nature of CMPT is so far still controversial. Recent genetic studies have revealed mutations in some driver oncogenes (*EGFR*, *BRAF*, *ALK*, and *KRAS*) and confirmed the concept that CMPT is a neoplastic disease as opposed to a reactive or metaplastic lesion.^9^, ^10^, ^11^ In addition, some studies have proposed that CMPT shows some pathological features of malignant potential, although it has ciliated epithelial cells.^12^ CMPT has a series of histological features (destruction of alveolar structures, proliferation along alveolar walls with skip lesions, no encapsulation and a micropapillary pattern) and mutations in driver oncogenes (*EGFR*, *BRAF*, *ALK* and *KRAS*), which supports the notion that the lesions tend to be neoplastic with malignant potential. Therefore, CMPT may be classified as a low‐grade malignant tumor. Although partial resection or segmentectomy might be sufficient for these low‐grade malignant or borderline tumors, pre‐ and intraoperative diagnosis is difficult and standard lobectomy remains the treatment of choice.^13^ In the case reported here, combined H&E staining and immunohistochemistry confirmed that this was mucinous adenocarcinoma caused by the cancerization of CMPT, which also provides histological evidence for the malignant potential.

**FIGURE 3:**
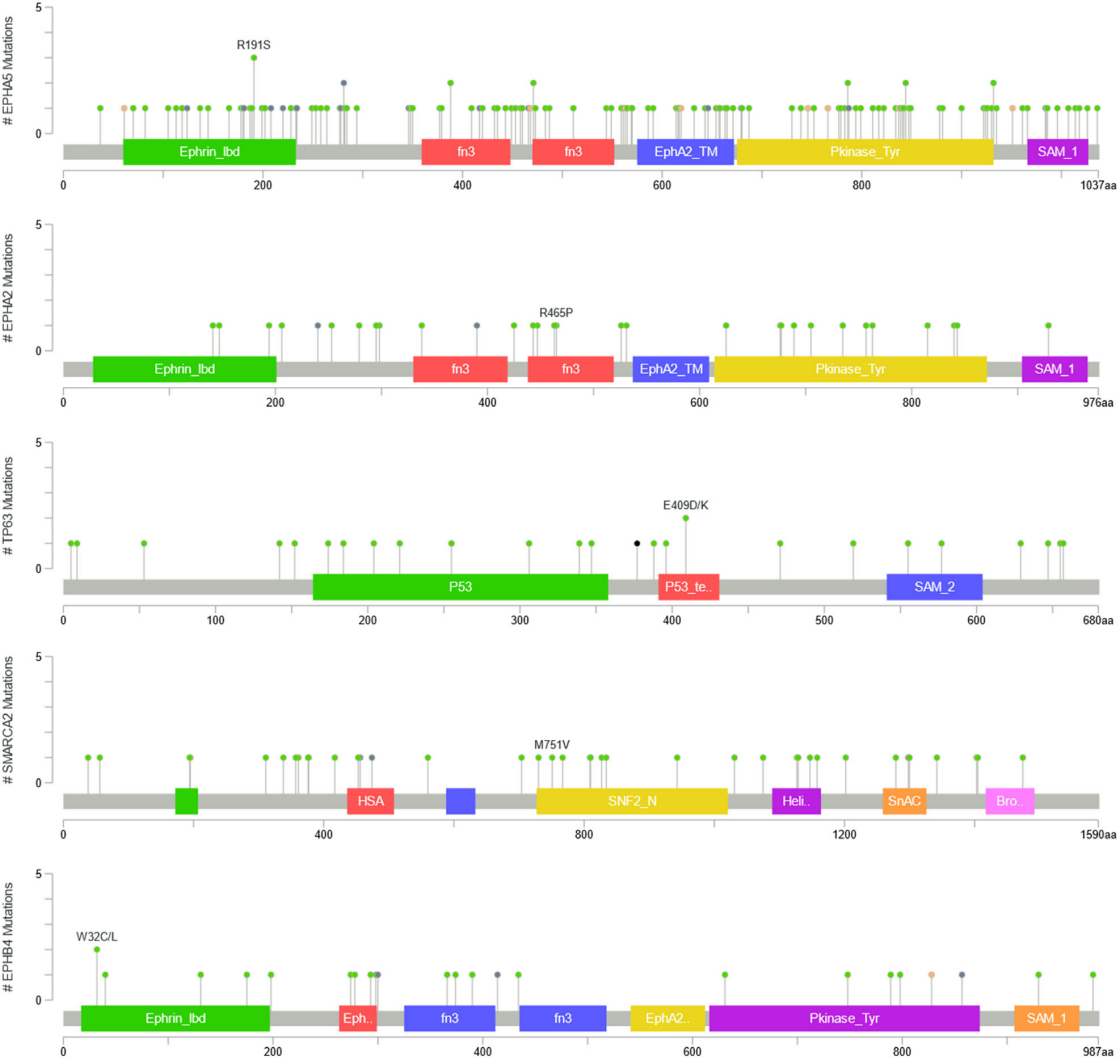
Gene mutation analysis results and corresponding data from The Cancer Genome Atlas (TCGA). The identified missense mutations in EPHA5, EPHA2, TP63, SMARCA2 and EPHB4 are overlaid with the mutation distribution diagrams of these genes from TCGA

Based on the limited data currently available, some studies have suggested that CMPT is the precursor of mucinous adenocarcinoma, analogous to the established AAH‐AIS‐adenocarcinoma sequence.^11^, ^14^ This may occur through a separate pathway independent of *EGFR*, *KRAS* or other known candidate genes involved in lung tumorigenesis, at least during the initial stages. In this case, there were some rare mutations found on next‐generation sequencing the tissue. The mutation distribution from TCGA of genes associated with lung cancer in the case reported here are highlighted in Figure 3. According to previous studies, EPHA5 mutation impairs natural killer (NK) cell‐mediated cytotoxicity against NSCLC cells and promotes migration and invasion in NSCLC cells.^15^ ERBB3,^16^ which is a therapeutic target for an antibody that is able to activate the complement system and mediate the antibody dependent cell‐mediated cytotoxicity to lyse the lung cancer cells, and EPHA2^17^ are promising targets for the treatment and prevention of NSCLC. In addition, previous studies have suggested that genetic variation in TP63 may influence lung cancer susceptibility in the Han population.^18^ Hypermethylation of SMARCA2 and EPHB4 may also play oncogenic roles in lung cancer development.^19^, ^20^

In conclusion, we report a right lung lower lobe GGO that was found in this patient on CT and diagnosed as mucinous adenocarcinoma caused by cancerization from CMPT. The case provides histological evidence for this hypothesis that CMPT can be the precursor of mucinous adenocarcinoma. More cases and further studies are also needed to confirm the genetic and histological nature of CMPT.

## CONFLICT OF INTEREST

All authors have declared no conflicts of interest.
